# The DO-KB knowledgebase 2026 update: expanding programmatic and language access

**DOI:** 10.1093/nar/gkaf1213

**Published:** 2025-11-26

**Authors:** J Allen Baron, Claudia M Sánchez-Beato Johnson, Michael A Schor, Dustin Olley, Lance Nickel, Victor Felix, Susan M Bello, Carol Greene, Richard Lichenstein, Katharine Bisordi, Rima Koka, Cynthia Bearer, Regina Macatangay, Nischal Ada, Kaitlin Ballenger, Emily Bliss, Lauren Colliver, Grace Dobbins, Harrison Heitzig, Shannan Dixon, Patrick Semesky, Jennifer Garth, Matthew Fairchild, Peter Gaskin, Sarina Zahid, Rachel Castillo, Sarah Edwards, Astrid Widjaja, Yamei Usui, Erin Lynch, Melissa Clarkson, Todd Detwiler, Lynn M Schriml

**Affiliations:** Institute for Genome Sciences, University of Maryland School of Medicine, Baltimore, MD 21201, United States; Institute for Genome Sciences, University of Maryland School of Medicine, Baltimore, MD 21201, United States; Institute for Genome Sciences, University of Maryland School of Medicine, Baltimore, MD 21201, United States; Institute for Genome Sciences, University of Maryland School of Medicine, Baltimore, MD 21201, United States; University of South Carolina, Department of Information Technology, Columbia, SC 29208, United States; Institute for Genome Sciences, University of Maryland School of Medicine, Baltimore, MD 21201, United States; Mouse Genome Informatics, The Jackson Laboratory, Bar Harbor, ME 04609, United States; University of Maryland School of Medicine, Baltimore, MD 21201, United States; University of Maryland School of Medicine, Baltimore, MD 21201, United States; University of Maryland School of Medicine, Baltimore, MD 21201, United States; University of Maryland School of Medicine, Baltimore, MD 21201, United States; Case Western Reserve University, School of Medicine, Cleveland, OH 44106, United States; University of Maryland School of Medicine, Baltimore, MD 21201, United States; University of Maryland School of Medicine, Baltimore, MD 21201, United States; University of Maryland School of Medicine, Baltimore, MD 21201, United States; University of Maryland School of Medicine, Baltimore, MD 21201, United States; University of Maryland School of Medicine, Baltimore, MD 21201, United States; University of Maryland School of Medicine, Baltimore, MD 21201, United States; University of Maryland School of Medicine, Baltimore, MD 21201, United States; University of Maryland School of Medicine, Baltimore, MD 21201, United States; University of Maryland School of Medicine, Baltimore, MD 21201, United States; University of Maryland School of Medicine, Baltimore, MD 21201, United States; University of Maryland School of Medicine, Baltimore, MD 21201, United States; University of Maryland School of Medicine, Baltimore, MD 21201, United States; University of Maryland School of Medicine, Baltimore, MD 21201, United States; University of Maryland School of Medicine, Baltimore, MD 21201, United States; University of Maryland School of Medicine, Baltimore, MD 21201, United States; University of Maryland School of Medicine, Baltimore, MD 21201, United States; University of Maryland School of Medicine, Baltimore, MD 21201, United States; University of Maryland School of Medicine, Baltimore, MD 21201, United States; University of Kentucky, College of Medicine, Lexington, KY 40506, United States; University of Kentucky, College of Medicine, Lexington, KY 40506, United States; Institute for Genome Sciences, University of Maryland School of Medicine, Baltimore, MD 21201, United States

## Abstract

The Human Disease Ontology Knowledgebase (DO-KB; https://disease-ontology.org/), a Global Core Biodata Resource, serves as a reference framework for multiscale biomedical data integration and analysis, within a unifying etiology-based disease classification. In this 2026 update of the Human Disease Ontology (DO) and DO-KB resource, we present significant advances in programmatic data retrieval and disease representation since our previous NARdb publication. Here we report on the development of a DO Nosology education program, the Spanish translation of the DO website and ontology content, and expansion of disease knowledge representation.

## Graphical Abstract

**Graphical Abstract:**
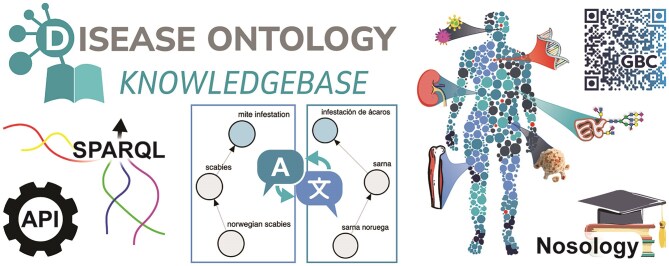

## Introduction

The Human Disease Ontology Knowledgebase (DO-KB; https://disease-ontology.org/) cadre of data discovery resources provides an integrated semantic system of human disease knowledge. Established in 2003, the Human Disease Ontology (DO) provides expertly curated, human-readable, and machine-actionable disease data within a comprehensive etiological-devised disease classification system of ∼12 000 genetic, infectious, cancer, environmental, complex, rare, and common diseases. The DO continues to address the challenges of an ever-expanding disease knowledge ecosystem integrating variable nomenclature representations represented across biomedical resources and augmenting the DO with advancing disease classification systems. Each month, the DO data release includes newly published disease terms, recently revised disease nomenclature and community-derived disease classifications. The DO is widely utilized as a disease data standardization resource, supporting a growing disease data knowledge graph. Integration of over 38 000 manually curated, (exact, broad, and narrow) cross-references provide a structured, connectivity framework between clinical vocabularies. DO-KB tools [e.g. DO-KB SPARQL Sandbox and SPARQL endpoint, Faceted Search tool, and advanced Application Programming Interface (API) service] provide programmatic data retrieval, along with enhanced data discovery empowered by the DO’s semantic knowledge, connecting disease-related data across Open Linked Data resources. SPARQL queries utilized for DO curation can be found in the DO-KB SPARQL queries repository (https://github.com/DiseaseOntology/SPARQLqueries).

Project development is driven to deliver the preservation and long-term availability of FAIR and TRUST-worthy disease knowledge [1, 2]. Advancing disease knowledge information extraction drives ongoing DO and DO-KB development. This, in turn, improves user capabilities for mining rare and complex disease data, towards advancing research and therapeutic efforts. Here we present the 2026 update of the DO and DO-KB, reporting on updates since our previous 2024 NAR database publication [3], highlighting DO content expansion of complex diseases, environmental drivers of disease, and disease re-classifications), enhancement of DO accessibility, development of Spanish DO, and augmented programmatic (SPARQL and API) access to DO’s semantic knowledge graph.

### Outreach

The DO-KB strategically conducts extensive outreach each year to expand our user community, to hear from stakeholders and to identify opportunities for collaboration and DO term expansion. DO outreach presentations are available via figshare (https://figshare.com/projects/Human_Disease_Ontology/124345) and via the DO’s YouTube channel (https://www.youtube.com/@DiseaseontologyOrgDOID/videos), with resource updates posted quarterly through the DO’s Newsletter (https://disease-ontology.org/community/newsletter/). In order to gain additional feedback from our user community, we launched this past spring our 2025 Annual User Survey, provided as a link at the top of DO website pages throughout 2025. We have established a public Slack channel to facilitate communication, which can be joined via the perpetual link (https://join.slack.com/t/humandiseaseontology/shared_invite/zt-25uhg4dyb-BLpcsVLhAqMFxfh∼hncPHA). In addition to X/Twitter, updates are also shared via BlueSky (@diseaseontology.bsky.social) https://bsky.app/profile/diseaseontology.bsky.social account.

### Global impact

The DO was designated a Global Core Biodata Resource, by the Global Biodata Coalition (https://globalbiodata.org/) in 2023, through a rigorous, global-peer review process, highlighting the DO’s fundamental role in global biomedical and clinical research, as a critical resource among the global infrastructure of interconnected resources. Quarterly, we identify and review the latest citations of DO project publications, referred to as our ‘cited by’ citations. To date, we have identified 2425 ‘cited by’ publications, reflecting a 25% increase (593 citations) since our last NARdb publication. This significant increase is greater than any prior period reported and highlights the increasing awareness and utility of DO disease connected data. A file of the DO ‘cited by’ citations is available in the GitHub/DOreports folder (publications_citing_DO.tsv). DO-KB and the DO continue to serve as a bridge that strengthens the links between disease data across the web and around the globe. Tracking the number of resources built that utilize the DO and their geographic location, we have noted an increase of 75 new resources in the past two years. To date, the DO has been integrated into >450 resources, developed in 48 countries (https://disease-ontology.org/community/use-cases/). The DO-KB website and services continue to be widely used by individual users, with >36 241 unique users from 156 countries accessing the website, as measured with GA4 Google Analytics since its implementation in July 2023. This represents nearly two thirds of the UN M49 standard “Countries or areas” (https://unstats.un.org/unsd/methodology/m49/) in the world today.

### Content expansion

Disease diagnostic complexity is challenged by phenotypic and molecular variability. The DO works to address this challenge by modeling complex disease etiology and integrating multiple molecular and environmental contributing factors into a singular framework. Since the DO’s previous NAR database publication (August 2023 release, v2023-08-08), the DO has expanded to include 11 946 disease classes, 9640 with textual definitions (80.7%)—an increase of 579 disease terms [607 new terms, 3 unobsoleted (a previously obsolete term is updated to active status), and 31 obsoleted disease terms] (August 2025 release, v2025-08-29). A file of these updates is available in our GitHub/DOreports folder (diseases_2023–25.tsv). DO release logs include a textual description of updated and added disease terms, obsoleted diseases and the release change log. Curation documentation files are shared via our public google docs folder, with links to this folder provided in the ‘Data availability’ section below [e.g. DO’s Simple Standard for Sharing Ontological Mapping (SSSOM) cross-mappings; NHGRI Curation, GlyGen term, ICTV viral infectious diseases, musculoskeletal and urogenital diseases, new/revised DO terms since 2023; and our viral infectious disease curation style guide]. Strategic growth into areas of evolving disease research has been instrumental in enhancing DO’s utility and enhancing stakeholder engagement. Here we highlight a number of key updates of note:

- **Online Mendelian Inheritance in Man (OMIM) prefix update:** the DO updated the cross-reference prefix for the OMIM (https://www.omim.org/) [4]. This work replaced the DO’s 5988 OMIM cross-reference prefix with the MIM prefix for all DO terms and the IRIs in the omim_susc_import (genetic susceptibilities). This work was conducted at the request of OMIM to align with OMIM’s preferred ID prefix (May 2024). Moving forward, all newly added OMIM cross references are represented with the MIM prefix.
- **Synonyms** that are considered **acronyms**, as defined in the new OBO Metadata Ontology annotation property for acronyms (https://github.com/information-artifact-ontology/ontology-metadata) (OMO:0003012), have been annotated as such. In order to identify synonyms that are acronyms (4020), an additional synonym type has been annotated to these synonyms. The acronyms can be retrieved via the all-synonyms-in-DO.rq SPARQL query that is included in the SPARQL Sandbox.
- **Language tags:** The utilization of language tags has been under discussion in the OBO Foundry [5] for several years (see GitHub issues: https://github.com/OBOFoundry/OBOFoundry.github.io/issues/479 and https://github.com/OBOFoundry/OBOFoundry.github.io/issues/325). While planning for the creation of a Spanish DO, we proactively decided to implement the inclusion of English language tags for the project’s ontology files. All language-specific text (labels, definitions, synonyms, and comments) in release files now includes a language tag (as defined in ISO 639; e.g. “en” or “es”) [6].
- **Cross-reference mappings** (aka: xrefs): Since its inception, the DO has integrated cross-references to other clinical vocabularies through manual review, to provide stable mappings between the DO and the vocabularies. These annotations are refined utilizing SKOS (Simple Knowledge Organization System) vocabulary match designations (close, exact, broader, related, or narrower) (https://www.w3.org/2009/08/skos-reference/skos.html). Periodically, for curation efforts, we now produce a disease to cross reference mapping table in SSSOM, to facilitate the exchange and integration of semantic entity mappings. To date, the DO includes 38 516 mappings (https://disease-ontology.org/about/statistics/), between DOIDs and MeSH, NCIThesaurus, OMIM, ICD9, ICD10_CM, ICDO, GARD, NORD, Orphanet, SNOMED_CT, and UMLS).
- **Definitions:** Each definition is manually researched and devised by a DO curator and annotated with sources of provenance. Periodically the status of these sources is programmatically checked in order to update definition sources, replacing no longer available sources and updated URL stems to reflect changes in source URLs. For example, all previous (and now defunct) Merriam Webster definition source URLs have now been updated to their latest, correct source.
- **DO enrichment from NHGRI resources:** DO’s content is enriched through the mining of disease content from genomic resources. For example, new disease terms, synonyms, and definition sources have been augmented with information represented on NHGRI resource pages. For example, adding the source link for Turner syndrome (https://research.nhgri.nih.gov/atlas/condition/turner-syndrome; DOID:3491) provides DO users with a direct link to view images from the Atlas of Human Malformation Syndromes [7] representing Turner syndrome in diverse populations. This work involved the systematic review of 66 terms from the Atlas of Human Malformation Syndromes, 49 terms from the Clinical Genomic Database [8], and three terms from the Red Cell Membrane Disorder Mutations Database [9]. This curation effort is recorded in the DO’s “NHGRI Curation” public google documents folder.
- **DO subsets:** Bespoke DO files, also referred to as “slims”, are included with each monthly DO data release. This update includes replacement of previously manually maintained slims to the automated creation of DO_NCIthesaurus_slim, with inclusion of all terms that have an NCI thesaurus xref, and DO_rare_slim, with inclusion defined by a GARD or Orphanet xref; and the addition of DO_childhood_cancer_slim (102 DO terms) developed for a NCI working group to include cancers that specifically develop during childhood.
- **Disease re-classification and updated nomenclature revisions:** Data representation have been enhanced via re-classification (e.g. for CAMRQ3/SCAR34, appendicitis, and childhood leukemias) and nomenclature revisions (e.g. for frontotemporal dementia and/or amyotrophic lateral sclerosis and Ohdo syndrome subtypes), identified via literature curation, to encompass the expanding corpus of disease knowledge. These revisions of several hundred DO disease terms, over the past 2 years, are noted in DO’s monthly release notes.

### Targeted knowledge enhancement

Highlights of targeted knowledge enhancement within the ontology includes glycosylation-related diseases, a full re-review of viral infectious disease branch, collaborative review of musculoskeletal, and urogenital diseases, along with enhancement of environmental and genetic driver ontologies and their associated diseases.

### Glycosylation-associated diseases

The DO team periodically conducts a systematic review of DO terms in resources utilizing the DO, to identify new diseases, as well as nomenclature and classification updates. For example, in collaboration with GlyGen [10], a periodic review of glycosylation associated DO terms was initiated via the retrieval of GlyGen’s disease terms (245 DO terms) via a federated SPARQL query. Manual review of this dataset identified 17 nomenclature updates, which were subsequently shared with our GlyGen collaborators.

### Viral infectious diseases (DOID:934)

The entire viral infectious disease branch was reviewed, with data for 129 of 135 DO diseases being revised. This work was spearheaded by a new collaboration with ICTV (International Committee on Taxonomy of Viruses; https://ictv.global/taxonomy/find_the_species) [11]. This work included the integration of the latest viral taxonomy information from NCBI taxonomy (https://www.ncbi.nlm.nih.gov/taxonomy) and ICTV taxonomy (https://ictv.global/taxonomy) for 101 DO terms (see March 2025 DO release notes for details). The review included updates to term names, definitions, definition sources, logical axioms, and xrefs. For this work, we developed an updated style guide for integrating viral nomenclature within the DO definitions. The NCBI taxonomy terms and identifiers are viewable in the DO’s disease term definitions and in the NCBI taxonomy (ncbitaxon) import, viewable in DO’s OWL tree.

### Musculoskeletal and urogenital diseases

The goal of this collaborative work has been to expand anatomy-to-disease mappings. We teamed up with Endless Forms Studio at the University of Kentucky (https://endlessforms.info) to begin mapping the DO to graphical libraries of anatomical phenotypes [12, 13] and investigate use of anatomical graphics to create graphical navigation for the DO. Beginning with diseases associated with a subset of the musculoskeleton, a proof-of-concept web application was developed that links regions of anatomical graphics with Uberon classes that are linked to DO diseases [14]. The web application is available at https://demo.endlessforms.info/do-browser/. Select the ‘left first costochondral joint to view an example DO link. In the DO we reviewed and updated the anatomical location of 1260 musculoskeletal system diseases. Additionally, we reviewed and updated the anatomical annotations, associated phenotypes and anatomical variants for 51 urogenital system and intersex-related diseases.

While developing this tool, we encountered an inherent limitation of OWL relationship modeling that prevented adding all possible diseases affecting a given anatomical location to the web application. As in most ontologies, the DO imports classes from other ontologies and defines disease-to-imported class relationships using relationship predicates, such as disease-to-anatomy relationships using the ‘disease has location’ (RO:0004026) predicate and Uberon anatomical entity classes. By design, OWL subclasses inherit the properties, including logical relationships, of their superclasses (a type of inference). Since ‘cartilage disease’ has the axiom ‘disease has location’ some ‘cartilage tissue’ (UBERON:0002418), it will be inferred that all its subclass descendants also affect cartilage tissue. This powerful approach allows reasoners to infer additional disease-to-disease relationships, and relationships between diseases and other classes, using the semantic definitions of a disease, all its superclass ancestors, and any imported classes.

It seems reasonable to assume that these logical relationships would be bidirectional: if a disease is related to a given anatomical location, one should be able to choose an anatomical location and identify all given diseases that affect it. This works for asserted logical axioms, those where a disease is directly linked to an anatomical location. Starting with the anatomical entity ‘costochondral joint’ (UBERON:0002293), the disease Tietze’s syndrome (DOID:14021) can be identified because the disease has the logical axiom ‘disease has location’ some ‘costochondral joint’ (a direct relationship). However, it is not always possible to model diseases with complete asserted relationships. For example, rheumatoid arthritis (DOID:7148) can affect many different joints, including the costochondral joints. Thus, it is appropriate to use the general axiom: ‘disease has location’ some ‘skeletal joint’ (UBERON:0000982) rather than defining an asserted relationship to each individual joint. Reasoners will rightly infer that all instances of rheumatoid arthritis affect one or more skeletal joints, however, this relationship does not imply that all skeletal joints are affected. In other words, the logical disease-to-anatomy relationship defines that a disease may affect a particular set of anatomical entities but the opposite, that all of these entities will be affected by a disease is not guaranteed. The relationship is not guaranteed to be bidirectional. Diseases defined with ‘disease has location’ some ‘skeletal joint’ make this evident. Rheumatoid arthritis has been known to affect costochondral joints and can be identified by walking up the anatomy hierarchy from ‘costochondral joint’ to ‘skeletal joint’ and then identifying diseases with either an asserted or inferred (from a superclass ancestor) ‘disease has location’ relationship to ‘skeletal joint’. However, this same approach also returns diseases that affect skeletal joints but do not affect the costochondral joints, including ‘craniosynostosis’ (DOID:2340) which has the asserted relationship ‘disease has location’ some ‘lambdoid suture’ (UBERON:0002491; a joint of the skull) since the lambdoid suture is a skeletal joint. From the anatomical perspective of these diseases, you can walk up the hierarchy and get some real relationships, but you are also likely to get spurious ones.

### Environmental and genetic drivers

The implementation of the DO’s environmental disease driver (DISDRIV) ontology has been publicly released (https://github.com/DiseaseOntology/DiseaseDriversOntology; https://obofoundry.org/ontology/disdriv.html). DISDRIV includes environmental, maternal, and social exposures, categorized into biological drivers, chemical drivers, ecological perturbations, nutrient deficiency, and socioeconomic drivers (with DISDRIV IDs). Specific drivers, classified under these categories, are defined in their source ontologies, NCBItaxon (https://github.com/obophenotype/ncbitaxon), OMIT (https://github.com/OmniSearch/omit), ENVO [15], NCIT (https://github.com/ncit-obo-org/ncit-obo-edition), FOODON [16], and ExO (https://github.com/CTDbase/exposure-ontology); for example, the environmental driver ‘alcohol’ is sourced from the ChEBI ontology [17].

Our University of Maryland School of Medicine Clinician team led literature review of diseases and their causal environmental drivers, with the identified driver-to-disease relationships being defined via DO logical axioms. For example, fetal alcohol syndrome (DOID:0050665) has been annotated with the axiom: ‘has disease driver’ some alcohol. Defining environmental drivers of human diseases is an ongoing area of work. DO disease terms are being annotated via logical axioms to define their relationship to environmental drivers, when the driver is documented in literature as a causal relationship. In the DO’s OWL tree viewer (https://disease-ontology.org/do/), DISDRIV annotated disease terms can be retrieved, via the Advanced Search, by selecting “Relation” and ‘has disease driver’. The Relation searches return diseases where the SubClassOf axioms for any of the thirteen Relation Ontology (RO) ‘relation’ terms [18]. This work is further informed by the recent examination of chemical drivers and biomarkers of neonatal exposures [19].

The DO’s omim_susceptibility import, defining genetic drivers of human diseases in the DO created in 2018, was revised in 2021 and re-reviewed in 2024 and 2025. Each genetic risk factor in the import, as defined in OMIM, was reviewed to identify description or classification updates. Likewise, OMIM susceptibility records were reviewed for new or revised descriptions. Nomenclature and disease-to-risk-factor associations were assessed and updated in the import to align with OMIM’s records (as of February 2025) (*N* = 266). The DO disease-to-genetic-risk-factors are defined by the axiom: ‘susceptibility to’ term from OMIM {RO relation} ‘contributes to condition’ some ‘disease’. For example, glioma susceptibility 1 (MIM:137800) ‘contributes to condition’ some ‘high grade glioma’ (DOID:3070).

### Nosology education program

Led by the DO’s Clinician team (co-authors Greene, Koka, Lichenstein, and Bisordi), we launched a clinical nosology education program in 2025, following the preparation of program materials in 2024 and the selection of our initial group of mentors. Course materials are available via the DO-KB Nosology web page (https://disease-ontology.org/resources/nosology/) with the Introductory and Curation slide decks available via the figshare Nosology Program collection.

We have devised a mentored, multidisciplinary team approach, to bring together clinical faculty mentors (physicians and genetic counselors) with medical, pharmacy, and genetic counselor trainees, with a focus on enhancing trainee appreciation of the importance of nosology. Clinician mentor and professional student mentee teams tackled disease classification challenges, enhancing their knowledge and advancing the DO’s disease representation. This educational project is designed to broaden trainees' understanding of the nuances and complexities of disease nosology, providing an opportunity to gain new perspectives on the classification of complex diseases and to advance cross-disciplinary nosology knowledge. This program illustrates how the organized knowledge in the DO is useful as a teaching tool for structured review and highlights the complexity of disease nosology.

Our first semester began with the selection of five clinician faculty mentors, each of whom was recruited and mentored by a member of DO’s Clinician Team. The faculty mentors recruited and led a total of thirteen mentees (group size ranging from two to five), as they explored the complexity of disease nomenclature. The program orientation, delivered to the mentors, provided an introduction to the DO, concepts of nosology and an outline of the planned training program. The trainees were introduced to the program via this recording, the curation guidelines presentation, mentor-mentee team meetings and small group meetings with the DO Team members. Following the trainee’s program orientation session, each mentor/mentee team selected an area of interest, proposed disease terms for review and conducted term classification and nomenclature reviews. Teams met at least monthly, reviewing the conditions in the DO and in the literature and proposing updates to the DO. The diseases reviewed for this iteration of the training program were: coarctation of the aorta, acrocapitofemoral dysplasia, avoidant/restrictive food intake disorder; Ehlers–Danlos syndrome and Ramsay Hunt syndrome. Reviews were collected in standardized reporting documents, reviewed by the DO Clinician Team and integrated into the DO. Updates included the addition of new disease terms, nomenclature and classification updates, revised definitions and cross-references. One outcome of the training program to highlight was the recognition (in the context of coarctation of the aorta) of the need for an organized approach, in which there is clinically recognized nomenclature for a condition after the intervention alters the anatomy and/or pathophysiology. The DO clinician team devised pre- and post-program surveys to determine participants’ knowledge of the field and the potential impact of the program. Subsequent review of the survey results noted an increase in knowledge on part of the faculty mentors and trainees and provided feedback for improving the next session (e.g. revised reporting form, additional curation sessions to discuss reviews). The Nosology education program will continue in subsequent semesters for students at the University of Maryland, Baltimore including the School of Medicine, Graduate School and Pharmacy, with plans for expansion to other schools in the future.

### Spanish DO

Biomedical ontologies have been primarily developed in English; however, there has been a growing interest in providing ontologies in multiple languages. The Human Phenotype Ontology and Mammalian Phenotype Ontology [20] have been among the first ontologies to provide their data in >1 language. These projects were developed through dedicated community translator volunteers or via the adoption of translations provided by collaborators. To further expand global use and promote internationalization of the Human Disease Ontology, we prototyped a workflow to translate the DO website and ontology data into Spanish, as Spanish is the second most often spoken language (13%) in the United States (https://www.census.gov/library/stories/2022/12/languages-we-speak-in-united-states.html). On a practical note, the DO team includes one native and one fluent Spanish speaking curator who conduct the manual translation reviews, identifying and revising translation variability.

### Spanish translation workflow

In order to fully translate the DO in short order and provide verified, high-quality translations, we devised a multistep protocol for translation, beginning with professional translation, which has been found to be less error prone than machine translation but not free from error [21], followed by semi-automated and manual review. Working with a professional Texas-based medical Spanish translation service (The Spanish Group, https://thespanishgroup.org/) to conduct the initial translations, we began by translating the DO-KB website content (30 883 words). This first step provided an opportunity to assess the provided translations for accuracy and completeness. We found the English to Spanish translations to be accurate and anecdotally similar to translations from Google Translate (which reportedly has >90% translation accuracy) [22, 23]. As the DO website is updated, new or revised English text is translated into Spanish as part of the monthly updates. Subsequently, we sent the translation service the labels, definitions, and synonyms for DO diseases classified in the Physical disorders branch (533 DO disease terms: 17 558 words; from release v2023-10-21). This iterative approach allowed us to: review and revise our workflow for this larger dataset; to enhance our translation review capacity; to test our protocol before translating the rest of the ontology; and to devise a baseline for judging automated review performance and identify potential errors, categorized as either character or word-based errors (Table 1).

**Table 1.** Character and word transformations for comparison

Group	Transformation	Examples
Character transformations	Case standardization	Upper (Syndrome) versus lower (syndrome) case
	Numeral standardization	arabic (type 5) versus roman (type V) numbers
	Diacritic standardization	Schönlein versus SchonleinHemorrágica brasileña versus hemorragica brasilena
	Space removal*(e.g. space [], tab [\t], return [\n])*	“PKD YS1” versus “PKDYS1”
	Punctuation removal*(e.g. period [.], comma [,])*	46,XX sex reversal 5 versus 46XX sex reversal 5
Word transformations	Tokenization (percent match)	Breast lobular carcinoma versus lobular breast carcinoma (100%)Daltonismo versus daltonismo rojo (50%)
	Word stemming	Ear, ears → earInhalación, inhalar → inhal
	Stopword removal	For English: e.g. to, in, ofFor Spanish: e.g. de, y, la
	Punctuation removal (word-split)*(e.g. period [.], comma [,])*	46,XX sex reversal 5 versus 46 XX sex reversal 5

The full set of character and word-based transformations applied to paired-English text (ontology text and Google Translate backtranslation) or paired-Spanish text (professional and Google Translate translations) in all possible combinations.

### Translation Quality Control (QC)

The automated, initial review of the provided translations was designed with three parts: (i) Google Translate translations; (ii) language-paired text comparison along with the calculation of a similarity score; and (iii) classification by comparison of the score against a cutoff. The translations by Google Translate included both forward translation of the original English ontology text to Spanish and the backtranslation of the professional Spanish translations to English. This produced two sets of English or Spanish paired text: one set in English—consisting of the original ontology text and Google Translate backtranslation of professional Spanish translation, and a second set in Spanish—consisting of the professional and Google Translate translations from the original English. We devised a similarity score that was calculated for each set, by first applying all possible combinations of character and word transformations (Table 1) to the language-paired text. The results that matched exactly or had the most words matching and required the least transformations resulted in the highest score. Character-based transformations are preferred over word transformations because they are less likely to alter meaning. The percent of words, in each transformed pair that exactly matched, and a complete similarity score were calculated according to the equation:


\begin{eqnarray*}
\textit{Score} = {{W}_{pct}} - \ {{W}_{pct}} \times \ \left( {t + {{W}_n} \times {{W}_{wt}} + {{C}_n} \times {{C}_{wt}}} \right)
\end{eqnarray*}

: percentage of matching words after any transformations (0–1 scale)

: word tokenization penalty

: number of word transformations applied

: word transformation weight

: number character transformations applied

: character transformation weight

Since character-based transformations generally represented minor translation errors, the penalty set was smaller (${{C}_{wt}}$= 0.02) than the penalty for word transformations (${{W}_{wt}}$= 0.05). Another distinct penalty was defined for word tokenization ($t$ = 0.15). All transformation penalties and the tokenization penalty scale by the percentage of words matching between the paired, transformed text to avoid generating negative scores and reduce the penalty effect relative to word matches. That is to say, the penalties themselves are scaled down (made smaller) when the percentage of matching word goes down. An example using the Spanish translation of “Ross River fever” (DOID:0050518) serves to demonstrate the calculation of the similarity score. The translation service’s translation was “fiebre del río de Ross” while Google Translate produced “Fiebre del río Ross”. Character transformations are not sufficient to make these exact matches because of the extra “de” in the professional translation. Instead, the best match included 100% of the words (${{W}_{pct}}$ = 1) after word tokenization ($t$ = 0.15), one word transformation (${{W}_n}$ = 1, ${{W}_{wt}}$ = 0.05; stopword removal of “de”), and one character transformation (${{C}_n}$ = 1, ${{C}_{wt}}$ = 0.02; case standardization), resulting in a final similarity score of 0.78 between the Spanish translations.

The highest score for each paired-language comparison, one for English and one for Spanish, were then averaged and compared against a cutoff score of 0.75. Final similarity scores equal to the maximum score of 1, which could only occur when both the English- and Spanish-paired texts were exact matches, were classified as “exact”. All other average scores above the cutoff were classified as “passed”, thus ready to add to the ontology with minimal or no review, and those at or below the cutoff were classified as “pending”, thus flagged for manual review prior to inclusion (Table 2).

**Table 2.** Translation matching categories

Text type	Classification category	Total	Percent
Labels	Exact	5197	45.62
	Passed	4630	40.64
	Pending	1566	13.75
Synonyms	Exact	6422	34.21
	Passed	8387	44.67
	Pending	3965	21.12
Definitions	Exact	20	0.22
	Passed	785	8.68
	Pending	8241	91.1

Total count and percentage of text elements classified as exact matches, passed automated scoring, or pending further review for each translated DO text type (labels, synonyms, definitions), to date.

Returning to the example of “Ross River fever” (DOID:0050518), the Google Translate backtranslation from the professional Spanish translation to English was “ross river fever” which was made exactly equivalent to the original English (${{W}_{pct}}$ = 1) with just one character-based transformation (${{C}_n}$ = 1, ${{C}_{wt}}$ = 0.02; case standardization) and no word-based transformations ($t$ = 0, ${{W}_n}$ = 0) resulting in a score of 0.98. Averaging the 0.98 (English) and 0.78 (Spanish) scores resulted in a final score of 0.88 which is above the 0.75 cutoff, so the professional Spanish translation was classified as “passed” and used as is in the ontology.

The code used to calculate these scores and manage and merge the various translation reviews in Google Sheets can be found in the DO_translation_es GitHub repository (https://github.com/DiseaseOntology/DO_translation_es). Penalty weights and the 0.75 cutoff were determined by optimizing the passing and failing translations between this automated score and the manually reviewed professional Spanish translations of the labels of the diseases in the physical disorder branch, with a strong preference against false positives.

The remaining branches of the DO (10 977 diseases totaling 332 039 words; release: v2024-02-28) were submitted to the translation service for professional translation. The automated scoring classification produced comparable results to the outcome of the Physical disorders branch comparison. In total, the complete scoring classification results of all ontology text, including the physical disorder branch, was of very high quality with >45% of the labels and 34% of synonyms being classified as “exact” (Table 2) and >40% of labels and synonyms exceeding our 0.75 high quality threshold (“passed”). This method drastically reduced the set of labels and synonyms needing further manual review. Definitions did not perform as well with this label-optimized scoring approach, likely due to their longer length and more inherent variability in English and Spanish phrasing.

### Data management and release

To add the reviewed Spanish translations to the ontology and identify text that changes over time, we initially explored using the Babelon (https://github.com/monarch-initiative/babelon) tool, a standard with supporting software for managing ontology translations and language profiles using simple TSV files that is being developed for HPO. However, the Babelon data model does not yet include features needed for this work (e.g. provenance for translation review, support for acronyms, or directly combining translations with existing ontology data). To address these challenges in our workflow, we reformatted translation data into TSV formats similar to those of Babelon, that include review provenance and acronym annotations. In order to track and manage ongoing DO text changes for translations, our workflow code compares text between the latest ontology data and translated records for an expanded set of text, including synonyms and ontology metadata annotations, and generates data subsets useful for specific translation tasks (https://github.com/DiseaseOntology/DO_translation_es/blob/main/scripts/prepare_release.R). Specifically, matches and changes are identified by comparing identifiers, predicates, and English text (triples). All records representing unique triples (id-predicate-English text), with accompanying translations and metadata, are sorted and saved in a single file (doid-es-all.tsv) to simplify future comparisons. Additional subsets of data are created as separate files to aid further translation and review, these files are available in the src/translations directory of the ontology’s GitHub repository (https://github.com/DiseaseOntology/HumanDiseaseOntology/tree/main/src/translations).

### Translated definitions

At this time, because definition scores perform poorly with the 0.75 label-optimized cutoff (Table 2), we have opted to include translation service’s definitions that fall below the 0.75 cutoff threshold in doid-es.tsv and ontology releases if they are not found to have obvious errors in a glancing review by a fluent team member.

### DO Spanish release workflow

Release pipelines were updated to convert the data in doid-es.tsv to templates designed for the addition of this information to the ontology with ROBOT, a well-established ontology-development automation software [24], and DO’s Makefile. The pipeline generates three versions of the ontology with translations at each release: an international version with all English and Spanish text (doid-international.owl) and two Spanish-specific versions (doid-es.owl and doid-es.obo). These files are updated with each monthly data release and are available in the src/ontology/releases/translations directory of the DO’s GitHub repository or by using their internationalized resource identifiers (IRIs; see the ‘Data availability’ section).

The initial release of translated DO content occurred in early 2025 (v2025-01-31) with additional translated content provided in subsequent monthly data release, as our team completes the manual review of the “pending” category and conducts definition translation reviews. To date (v2025-08-29), Spanish DO include: 70.72% (28 941/40 921) of the total DO text; with 92.73% (11 078/11 946) of the DO labels, 59.28% (11 461/19 335) of DO synonyms and 66.41% (6402/9640) of DO definitions. A new DO-KB translation page (https://disease-ontology.org/resources/translation/) provides an overview of translation efforts, while detailed information about accessing ontology files can be found in README-release-files.md (https://github.com/DiseaseOntology/HumanDiseaseOntology/blob/main/src/translations/README-translation.md). Additional documentation of this work has also been provided via a figshare presentation (https://doi.org/10.6084/m9.figshare.28840925.v1) and YouTube video (The Spanish Translation of the Disease Ontology; https://youtu.be/mfg42Vc0Vn4?si=oyz1Jsr2uAD-JSOY).

## DO-KB infrastructure development

### Programmatic data retrieval

A focus on modernizing infrastructure is imperative to provide new mechanisms for data interoperability and accessibility. Our strategic approach includes: following community best practices (e.g. OBO Foundry, FAIR principles); adapting established technical approaches (e.g. Neo4j; following OpenAPI specifications); and openly sharing project-developed tooling. Thus, reducing technical debt while maximizing data delivery opportunities. The DO-KB tools (e.g. DO-KB SPARQL service, Faceted Search Interface, and advanced API service) enhance data discovery, delivering an integrated data system that exposes the DO’s semantic knowledge and connects disease-related data across Open Linked Data resources.

### SPARQL sandbox

Enhancing the user experience, the **SPARQL Sandbox** results table is now paginated, with the number of results (rows) shown below the table. Quarterly, additional SPARQL queries are added to the Sandbox, which has an expanded set of available queries (increased from 15 to 55). Organizationally, we have implemented a folder system to group SPARQL queries (Datasets, Federated, General, Imports, and Metadata). A prefixes.rq SPARQL has been added to provide frequently used prefixes for building queries. Example queries that explore disease-linked data in other resources beyond the DO, called federated queries, have been added to retrieve: (i) cancer proteoforms associated with DO disease terms in the Protein Ontology [25]; (ii) endocrine disease cell lines from Cellosaurus; and (iii) lipid metabolism disorders associated with pathways in Wikipathways. Updates to the set of provided queries are integrated into the site monthly from our GitHub repository (https://github.com/DiseaseOntology/SPARQLqueries). Documentation has been added to direct users to use the SPARQL endpoint (https://sparql.disease-ontology.org/) for large queries. The SPARQL Sandbox is a handy tool for exploring DO content. For example, to look up how many terms are in the omim_susceptibility import, one can go to the SPARQL sandbox, select the import folder, select the SYMP query, and replace ‘SYMP’ with ‘MIM’, to retrieve the list of OMIM susceptibility entities (count at the bottom of the page = 266).

### Application Programming Interface

We have developed an OpenAPI Specification (OAS) 3.1 compliant API. Being OAS compliant means that the DO-KB API is not only a RESTful system, but one which conforms to the most widely adopted API standards. This allows consumers of the API to more easily understand the API definition and develop client applications against it. The API specification is hosted on SwaggerHub (https://app.swaggerhub.com/apis/UMIGS/DiseaseOntology). Additionally, we have added a new API documentation page (https://disease-ontology.org/do-kb/api_doc/) on the DO-KB portion of the website where we host an interactive development page which is powered by SmartBear’s SwaggerUI (https://swagger.io/).

While the DO previously had a single API endpoint for returning term data, end users were required to first have a DOID available in order to use the endpoint. The new API allows users to access eight distinct endpoints in order to mine the DO data in a much more robust way. At our new /terms/search endpoint, a user can combine all of the functionality of our site’s Advanced Search with that of our Faceted Search. For example, using a curl command, a user may now search for all DO terms whose name contains “dermatitis” and are related to the UBERON term “epithelium” by sending the following query to the aforementioned endpoint as follows:

curl -s -X POST -H “Content-Type: application/json” “https://api.disease-ontology.org/v1/terms/search?page=1&page_size=50” –data @- << EOF

{

“data” : {

“names” : [“dermatitis”]

},

“imports” : {

“anatomy” : [“epithelium”]

}

}

EOF

In addition to the endpoint described above, the remaining (seven) endpoints allow users to: retrieve specific terms based on ID, label, or simply all DO terms; retrieve general information about the API version and data build itself; and finally search or retrieve imported ontology terms. The data returned by this new API for each term has also been substantially expanded.

## DO website expansion

A number of new web pages have been added to enhance the user experience (e.g. Registry of Contributors, Nosology program, and DO Translation for DO Spanish resources). We have enhanced the website search features and are providing new capabilities for programmatic data retrieval (e.g. API and Federated SPARQL queries), as outlined below.

### DO-KB home page

The DO-KB project landing page has been redesigned, with the addition of a rotating banner and five large navigational tiles which allow users to: quickly jump to our programmatic tools; jump to the DO ontology tree view; or submit a new term request via GitHub. Above those tiles, a permanent ontology search box has been added, to directly submit a query and look up ontology terms. When a search is submitted, the search results are returned on the tree visualization and search page (aka: DO ontology tree view).

The tree visualization itself has also been improved. The OWL and OBO trees are now available in Spanish, and users can search the Spanish translations of terms as well, after selecting the ‘español’ button, at the top of the page. This new **‘language selector’** (teal-colored español/English) button, which toggles between English and Spanish, changes both the webpage text language, handled by the Python Babel library (https://github.com/python-babel/babel), and the language of ontology data in the OBO and OWL tree browsers and supporting search bars, handled with server-side logic. On the backend side of this feature, we now store Spanish and English separately in our Neo4j graph database. Each language has its own OWL and OBO graph, so in total there are four graphs being maintained.

### Contributor resources

The contributions of scientific researchers, clinical practitioners, and organizations are invaluable to the development and maintenance of the Human Disease Ontology. The collective knowledge, perspectives, and expertise from this broad community ensure the ontology remains comprehensive, accurate, and up to date. An ORCID ID based attribution page, **Registry of Contributors** (https://disease-ontology.org/community/contributors/), has been developed to recognize and celebrate the invaluable contributions by individuals and organizations over the past two decades. The registry has been populated through the mining of internal documentation, GitHub issue requests and collaborator initiatives. To date, the registry includes 71 individual and 19 resource/group contributors. We developed and shared a DO-KB ‘**Code of Conduct’** and documented our ‘**Contributor Guidelines**’ at the top level (https://github.com/DiseaseOntology/HumanDiseaseOntology/tree/main) of our GitHub repository to communicate clear expectations and guidelines with our user community.

### Advanced search—DO OBO and OWL trees

In the Advanced search box, available on the DO tree browser page, search capabilities have been augmented to enable queries of logical axioms via compound searches of the DO’s 12 RO terms and a disease term. When the ‘relation’ option is selected, users are prompted to select a ‘Relation’ and a Keyword. For example, users can explore annotations for diseases that are a component of another disease by (i) selecting a RO term ‘disease has feature’ and (ii) adding a space in the Keyword search box. This query returns 95 DO records with 250 ‘disease has feature’ axioms, where one disease is a feature of another disease. For example, one of the returned results identifies invasive aspergillosis (DOID:0050073) which includes the logical axiom: ‘disease has feature’ some pneumonia. Note there are several examples where >1 ‘disease has feature’ axiom is annotated to a single DO term. Another query, ‘disease has driver’ retrieves 18 disease terms containing 26 annotations. For example, ‘fetal alcohol syndrome’ (DOID:0050665) includes the logical axiom: ‘has disease driver’ some alcohol (CHEBI:30879), as defined in DISDRIV.

### Updated graph visualization

**Figure 1.**
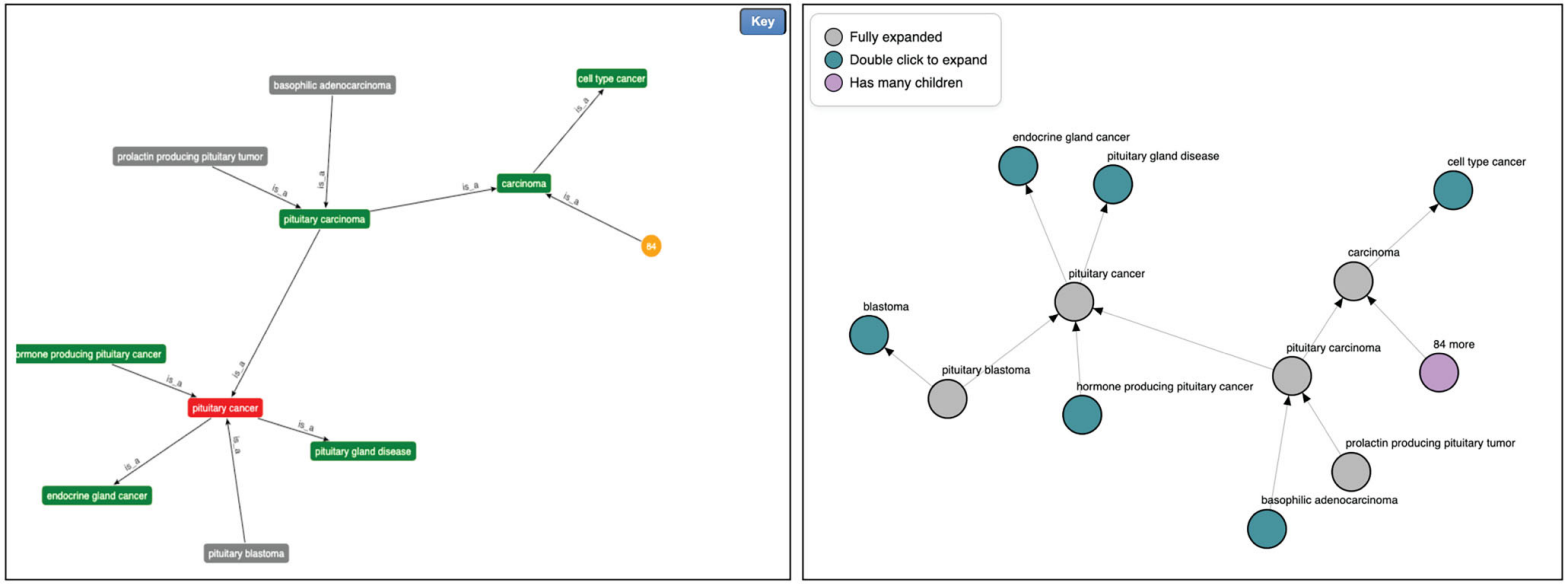
DO-KB Visualization page comparison. The prior visualization page (left) versus new page (right).

When navigating the DO, we have historically supported the ability to see the graph’s visual representation by clicking the “Visualize” button on a specific term definition page. The original library utilized to build the visualization, Arbor.js, was a valuable tool for dynamic graph visualization in the early 2010s. However, it has lacked updates in recent years which has left its visual appearance to become dated and its feature set to have been far superseded by more modern and comprehensive libraries (Fig. 1). We selected D3.js (https://d3js.org/), an industry standard for web visualizations, to develop a revamped and more interactive visualization system. Our new visualization page has a modern, cleaner look and feel which enables users to more easily digest term relationships. It has also been enhanced with new functionality to: allow users to pin terms within the view; to double-click in order to collapse nodes that are no longer of interest; to hover to see tooltips; and to pan and zoom.

The website’s backend updates include an upgraded graph database, from Neo4j version 3 to 5.26 (https://neo4j.com/). This change provides better performance, stability, and long-term support. We also added Google Analytics event logging in two places: server-side logging on the faceted search and SPARQL query endpoints; and browser-based logging on the SPARQL sandbox and faceted search webpages. This lets us see how people are actually using these tools so we can keep improving them. Finally, we added a robots.txt file and two sitemaps identifying static webpages and disease-specific pages to keep automated bots from crawling the site unnecessarily. This change helps cut down on wasted resource usage and keeps things running more smoothly for actual users.

## Future directions

Augmenting the DO content is a major area of focus for the project, as hundreds of disease terms are published or revised in literature each year. This work will include identifying revisions to previous areas of curation, for example, furthering the classification of red cell membrane disorders through additional literature review [26] and continuing our clinician-led review of overlap syndromes. Now that the workflow for identifying and translating new DO content into Spanish is in place, our curation translation work will keep apace with our monthly releases. Review of the translations of our other ontologies [Symptom Ontology (SYMP), Pathogen Transmission Ontology (TRANS), and Disease Drivers (DISDRIV)] are slated for near term areas of work. In the future, we plan to explore incorporating translations into more disease-ontology.org services (e.g. API, SPARQL, faceted search). Keep an eye out for additions to our “Advanced search” features, to include the omim_susceptiblity (genetic risk factor) query: ‘contributes to condition’ some ‘DO disease’ and for further annotations of environmental drivers of human diseases via additional annotations in DO with DISDRIV terms.

## Data Availability

All data and code produced for this work is made available under the Creative Commons Zero v1.0 Universal (CC0) license (https://creativecommons.org). Data files and code are available from the project’s GitHub repository (https://github.com/DiseaseOntology/) and DO_translation_es repository. Our monthly releases are also provided via Zenodo (e.g. August 2025: https://zenodo.org/records/16996094). As noted above, here are links to mentioned GitHub files, Google documents, and presentations: DO term updates: https://github.com/DiseaseOntology/HumanDiseaseOntology/blob/main/DOreports/diseases_2023-25.tsv DO Reports GitHub folder: https://github.com/DiseaseOntology/HumanDiseaseOntology/tree/main/DOreports DO’s public google docs (curation): https://drive.google.com/drive/u/0/folders/1zVxpziolifR9xOXG2xizfLhUr9mRtQBt Nosology Program figshare collection: https://doi.org/10.6084/m9.figshare.c.8005666.v1 Ontology files including translations can be accessed using their IRIs. OWL files are in RDF/XML. doid-international.owl: http://purl.obolibrary.org/obo/doid/translations/doid-international.owl doid-es.owl: http://purl.obolibrary.org/obo/doid/translations/doid-es.owl doid-es.obo: http://purl.obolibrary.org/obo/doid/translations/doid-es.obo Code for management of DO translation review and comparison with existing ontology data: https://github.com/DiseaseOntology/DO_translation_es Please use ontology IRIs to stably access or download ontology files. Specific ontology files mentioned in this publication include those from the: 1. **Human Disease Ontology** doid.owl (primary release product) – https://purl.obolibrary.org/obo/doid.owl doid-international.owl – https://purl.obolibrary.org/obo/doid/translations/doid-international.owl doid-es.owl – https://purl.obolibrary.org/obo/doid/translations/doid-es.owl doid-es.obo – https://purl.obolibrary.org/obo/doid/translations/doid-es.obo 2. **Disease Drivers Ontology** disdriv.owl – http://purl.obolibrary.org/obo/disdriv.owl Ontology release files, along with supporting files, can also be accessed from their corresponding GitHub repositories: 1. **Human Disease Ontology repository** – https://github.com/DiseaseOntology/HumanDiseaseOntology All release products – https://github.com/DiseaseOntology/HumanDiseaseOntology/tree/main/src/ontology/releases Translation source files (e.g. README-translation.md, doid-es-all.tsv) – https://github.com/DiseaseOntology/HumanDiseaseOntology/tree/main/src/translations DOreports (e.g. publications_citing_DO.tsv, diseases_2023-25.tsv) – https://github.com/DiseaseOntology/HumanDiseaseOntology/tree/main/DOreports OMIM susceptibility import – https://github.com/DiseaseOntology/HumanDiseaseOntology/blob/main/src/ontology/imports/omim_susc_import.owl 2. **Disease Drivers Ontology repository** – https://github.com/DiseaseOntology/DiseaseDriversOntology Release product – https://github.com/DiseaseOntology/DiseaseDriversOntology/blob/main/src/ontology/disdriv.owl Additional GitHub repositories supporting this publication include: 1. **DO_translation_es** for code used to manage translation data, execute automated review, and prepare Spanish DO release source files – https://github.com/DiseaseOntology/DO_translation_es Script that prepares translation data for release – https://github.com/DiseaseOntology/DO_translation_es/blob/main/scripts/prepare_release.R 2. **SPARQL queries** for queries supporting the DO-KB SPARQL Sandbox – https://github.com/DiseaseOntology/SPARQLqueries Files supporting curation can be found in a public Google Docs folder – https://drive.google.com/drive/folders/1zVxpziolifR9xOXG2xizfLhUr9mRtQBt public_Curation_style_guide-Viral_diseases.gdoc public_disease_viruses_ICTV_review.gsheet public_disease-musculoskeletal location in DO.xlsx public_do_sparql_results-query_Glygen.gsheet public_DOID_SSSOM_mappings.gsheet public_NHGRI resources diseases_review.gsheet public_urogenital_anatomy_disease_review.gsheet Outreach and Informative Products created and mentioned in this publication are available from: 1. The DO public figshare project – https://figshare.com/projects/Human_Disease_Ontology/124345 Nosology Program collection – https://doi.org/10.6084/m9.figshare.c.8005666.v1 Spanish DO – https://doi.org/10.6084/m9.figshare.28840925.v1 2. The DO public YouTube channel – https://www.youtube.com/@DiseaseontologyOrgDOID The Spanish Translation of the Disease Ontology – https://youtu.be/mfg42Vc0Vn4?si=oyz1Jsr2uAD-JSOY
